# 285. The Pediatric Urinary Microbiome: A Comparison of infants under 12 months with and without Urinary Tract Infection in South Korea

**DOI:** 10.1093/ofid/ofad500.357

**Published:** 2023-11-27

**Authors:** Ji Hyen Lee, Jung Won Lee

**Affiliations:** Ewha Womans University, College of Medicine, Department of Pediatrics, Seoul, Seoul-t'ukpyolsi, Republic of Korea; Ewha Womans University, College of Medicine, Department of Pediatrics, Seoul, Seoul-t'ukpyolsi, Republic of Korea

## Abstract

**Background:**

The microbiome of the urinary tract plays a significant role in maintaining health through its impact on bladder homeostasis. Urobiota helps maintain the urothelial integrity and prevents urinary tract infection (UTI), regulating neurotransmission and promoting local immune function. However, the number of studies on the pediatric population, especially those in the infant age group in Asia, is limited.

**Methods:**

We prospectively analyzed the urine microbiome of 15 Infants under 12 months between Jan. 2022 and Dec. 2022, grouped as UTI and control groups. UTI group was defined with fever (body temperature ≥38°C), pyuria (≥5 WBC/ high power field), significant bacteriuria (positive urine culture with pure growth 50,000 colony forming unit (CFU)/mL in catheterized urine culture) and control group was recruited from healthy infants who came to our hospital for vaccination but were uninfected. The urine samples underwent DNA extraction and 16S ribosomal RNA gene sequencing, urinalysis and urine culture. The Shannon diversity, the relative abundances of genus and species level, was analyzed with a Mann-Whitney U test and Kruskal-Wallis test.

**Results:**

The number of subjects was fifty and the mean age of the pediatric subjects was 6.2 months old. Ten of the subjects were in the control group (the ratio of male to female was 5:5), and five of the subjects in the UTI group (the ratio of male to female was 2:3). Most common genus are *streptococcus, bacteroids, prevotella, citrobacter,* and *bifidobacterium* in control group, while UTI group are frequently dominated by the genus *Escherichia coli, streptoccus, bacteroids, prevotella*, and *bifidobacterium*. There was no difference in genus lactobacillus between the two groups. In the analysis of the Shannon diversity, those with UTIs had significantly decreased diversity compared to the control group (*P* =0.037).

The top 20 genus in pediatric urine specimens

**Figure:**
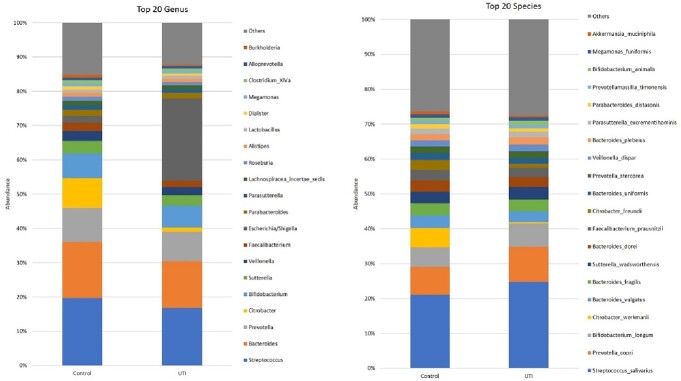

The comparison of Shannon diversity of control group and pediatric Urinary tract infection group in South Korea

**Figure:**
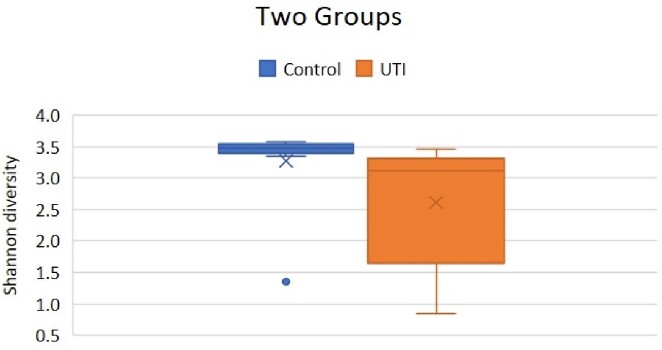

**Conclusion:**

A urinary microbiome was identified in infants under 12 months and we observed the differences in microbiome diversity and composition. The pediatric urinary microbiome has just begun to be explored, and further research and investigation must be conducted into about different ethic and demographic pediatric population characteristics.

**Disclosures:**

**All Authors**: No reported disclosures

